# Regulatory mechanism of circular RNAs in neurodegenerative diseases

**DOI:** 10.1111/cns.14499

**Published:** 2023-10-21

**Authors:** Feng Xiao, Zhi He, Siqi Wang, Jiamei Li, Xiaolan Fan, Taiming Yan, Mingyao Yang, Deying Yang

**Affiliations:** ^1^ College of Animal Science and Technology Sichuan Agricultural University Chengdu China; ^2^ Farm Animal Genetic Resources Exploration and Innovation Key Laboratory of Sichuan Province Sichuan Agricultural University Chengdu China

**Keywords:** biomarkers, circular RNAs, neurodegenerative diseases, pathogenesis, regulatory mechanism

## Abstract

**Background:**

Neurodegenerative disease is a collective term for a category of diseases that are caused by neuronal dysfunction, such as Alzheimer's disease (AD), Parkinson's disease (PD), and amyotrophic lateral sclerosis (ALS). Circular RNAs (circRNAs) are a class of non‐coding RNAs without the 3′ cap and 5′ poly(A) and are linked by covalent bonds. CircRNAs are highly expressed in brain neurons and can regulate the pathological process of neurodegenerative diseases by affecting the levels of various deposition proteins.

**Aims:**

This review is aiming to suggest that the majority of circRNAs influence neurodegenerative pathologies mainly by affecting the abnormal deposition of proteins in neurodegenerative diseases.

**Methods:**

We systematically summarized the pathological features of neurodegenerative diseases and the regulatory mechanisms of circRNAs in various types of neurodegenerative diseases.

**Results:**

Neurodegenerative disease main features include intercellular ubiquitin–proteasome system abnormalities, changes in cytoskeletal proteins, and the continuous deposition of insoluble protein fragments and inclusion bodies in the cytoplasm or nucleus, resulting in impairment of the normal physiological processes of the neuronal system. CircRNAs have multiple mechanisms, such as acting as microRNA sponges, binding to proteins, and regulating transcription. CircRNAs, which are highly stable molecules, are expected to be potential biomarkers for the pathological detection of neurodegenerative diseases such as AD and PD.

**Conclusions:**

In this review, we describe the regulatory roles and mechanisms of circRNAs in neurodegenerative diseases and aim to employ circRNAs as biomarkers for the diagnosis and treatment of neurodegenerative diseases.

## Graphical abstract

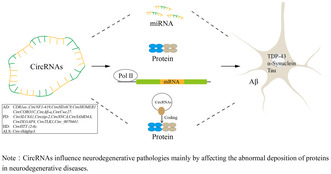

## 1 INTRODUCTION

Neurodegenerative diseases are slow‐progressing diseases caused by progressive damage and selective dysfunction of neurons in the central and peripheral nervous system, with an age of onset usually between 50 and 70 years.^1^ The main pathogenesis mechanisms of neurodegenerative diseases included abnormalities in the intercellular ubiquitin‐proteasome system, changes in cytoskeletal proteins, and continuous deposition of insoluble protein inclusion bodies in the cytoplasm or nucleus, such as β‐amyloid deposition, neurofibrillary degeneration, and Lewy body formation.^2^ These factors lead to subsequent pathological changes, including oxidative stress, neuroinflammation, abnormal autophagosome/lysosomal system, and programmed cell death.^1^

Circular RNAs (circRNAs) are a class of small‐molecule noncoding RNAs (ncRNAs) with special biological functions that are widely expressed in the cells, tissues, and organs of multiple species,^3^ such as *Homo sapiens,* ^4^ *Mus musculus,* ^5^ *Caenorhabditis elegans,* ^6^ and *Drosophila melanogaster*.^7^ CircRNAs were enriched in the synapses of neurons^8^, ^9^, ^10^ and regulated the pathological process of neurodegenerative diseases via various signaling pathways, including the nuclear factor kappa‐B (NF‐κB) signaling pathway,^11^ Wnt/β‐catenin pathway,^12^ and mitogen‐activated protein kinase (MAPK) pathway.^13^

This review discusses the pathogenesis of neurodegenerative diseases and circRNAs in four major neurodegenerative diseases with the aim of laying the groundwork for exploring circRNAs as biomarkers for the diagnosis and treatment of age‐related neurodegenerative diseases.

## 2 NEURODEGENERATIVE DISEASES AND PATHOGENIC FACTORS

### 2.1 Classification of neurodegenerative diseases

Several criteria, including clinical symptoms, anatomical region of neuronal dysfunction, and major molecular or protein conformational variants, had been utilized to classify neurodegenerative diseases^1^, ^14^, ^15^ (Table 1). For example, based on clinical symptoms, neurodegenerative diseases could be classified as Alzheimer's disease (AD), Parkinson's disease (PD), Huntington's disease (HD), and amyotrophic lateral sclerosis (ALS). In addition, neurodegenerative diseases can also be named amyloidosis, tauopathy, alpha‐synucleopathy, and transactivation response DNA‐binding protein 43 (TDP‐43) proteinopathy according to the major molecular or protein conformational variants. These major molecules and proteins have specific conformational properties that influence neurodegenerative diseases.

**TABLE 1:** Classification of neurodegenerative diseases.

Classify	Disease types
Clinical symptoms	Alzheimer's disease (AD), Parkinson's disease (PD), Huntington's disease (HD), and amyotrophic lateral sclerosis (ALS)
Anatomical region of neuronal dysfunction	Frontotemporal degenerative diseases, extrapyramidal diseases, and spinocerebellar degenerations
Major molecular or protein conformational variants	Amyloidosis, tauopathy, alpha‐synucleopathies, and transactivation response DNA‐binding protein 43(TDP‐43) proteinopathy

### 2.2 Aging and neurodegenerative diseases

Aging refers to a series of degenerative changes that occur in tissues, organs, and the whole body over time as the body matures and cell function gradually declines and eventually dies.^16^, ^17^ Various pathogenic factors of neurodegenerative diseases were closely related to the aging process, such as abnormal deposition of proteins, DNA damage, mitochondrial dysfunction, cellular aging, metabolic dysfunction, dysregulation of nicotinamide adenine dinucleotide (NAD+) levels, oxidative stress, stress response, telomerase inactivation, and inflammation.^18^ Some of these features have been observed in AD and PD.^18^, ^19^ Then, exogenous administration of NAD+ could prolong the lifespan of *C. elegans* and improve the pathogenesis and pathological characteristics of age‐related neurodegenerative diseases.^20^ Furthermore, the inhibition of mTOR signaling by rapamycin enhanced neuroprotection and inhibited cellular senescence.^21^ Therefore, exploring the link between neurodegenerative diseases and aging characteristics may lead to new therapeutic strategies for such diseases.

### 2.3 Abnormal deposition of proteins

**FIGURE 1:**
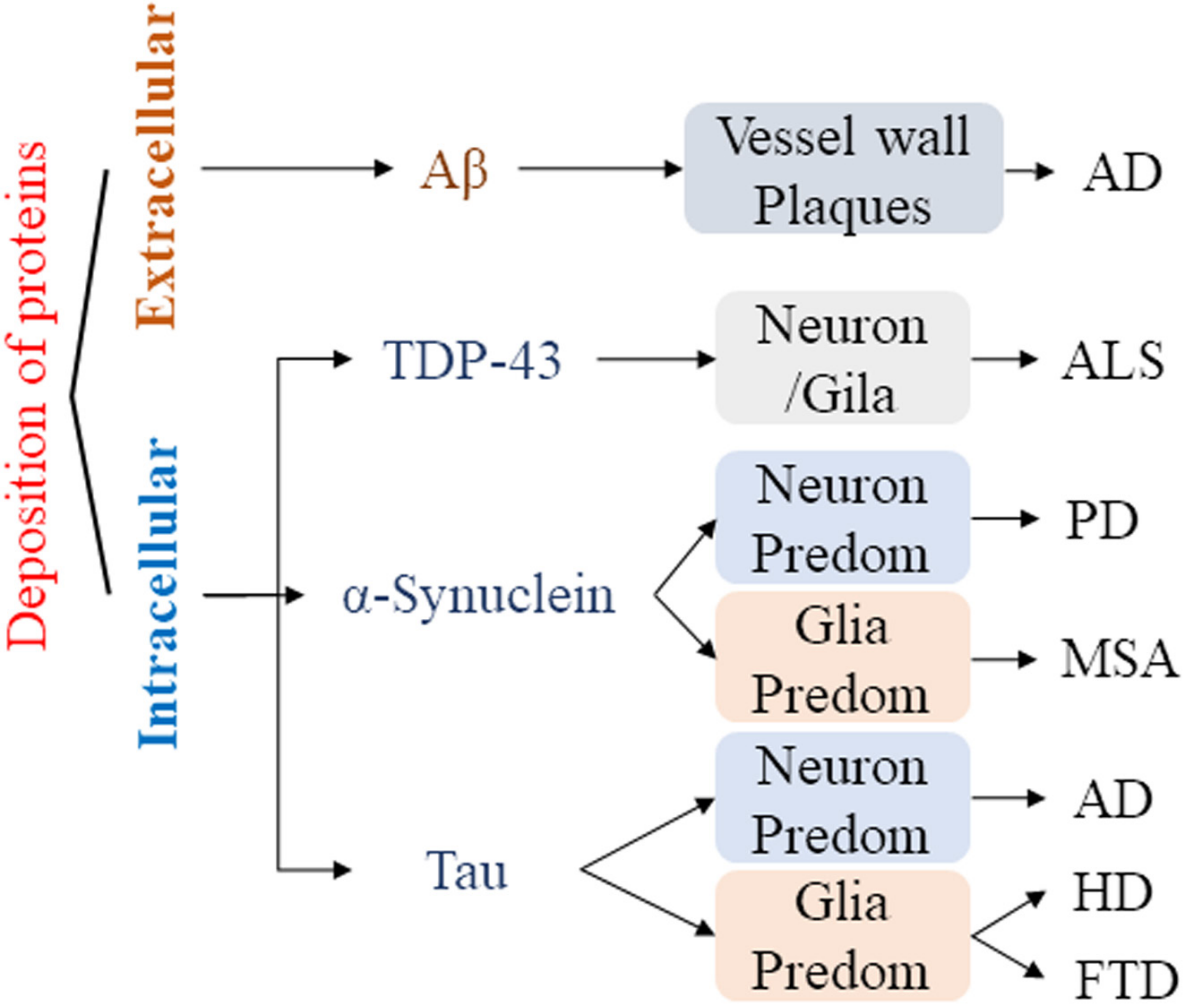
Protein deposits in various neurodegenerative diseases. AD, Alzheimer's disease; HD, Huntington's disease; PD, Parkinson's disease; ALS, amyotrophic lateral sclerosis; MSA, Multiple System Atrophy; FTD, Frontotemporal dementia.

Neurodegenerative diseases are characterized by progressive damage and selective dysfunction of neurons, associated with pathologically altered proteins deposited in the human brain as well as in peripheral organs. Abnormal conformational protein deposition impaired normal physiological processes of the neuronal system.^22^ These proteins included the β‐amyloid protein (Aβ), tauopathies, synuclein‐alpha (SNCA), and TDP‐43.^23^ The deposition of abnormal proteins affected the function of neurons in brain tissue to varying degrees, resulting in cognitive and functional impairments^24^ (Figure 1). Although biochemical modification of proteins is a potential therapeutic target and biomarker in neurodegenerative diseases, there is currently no effective clinical method for accurately identifying inclusion bodies formed by abnormal proteins in the course of neurodegenerative diseases.

## 3 BIOGENESIS OF CIRCRNAS IN NEURODEGENERATIVE DISEASES

### 3.1 Function of circRNAs

**FIGURE 2:**
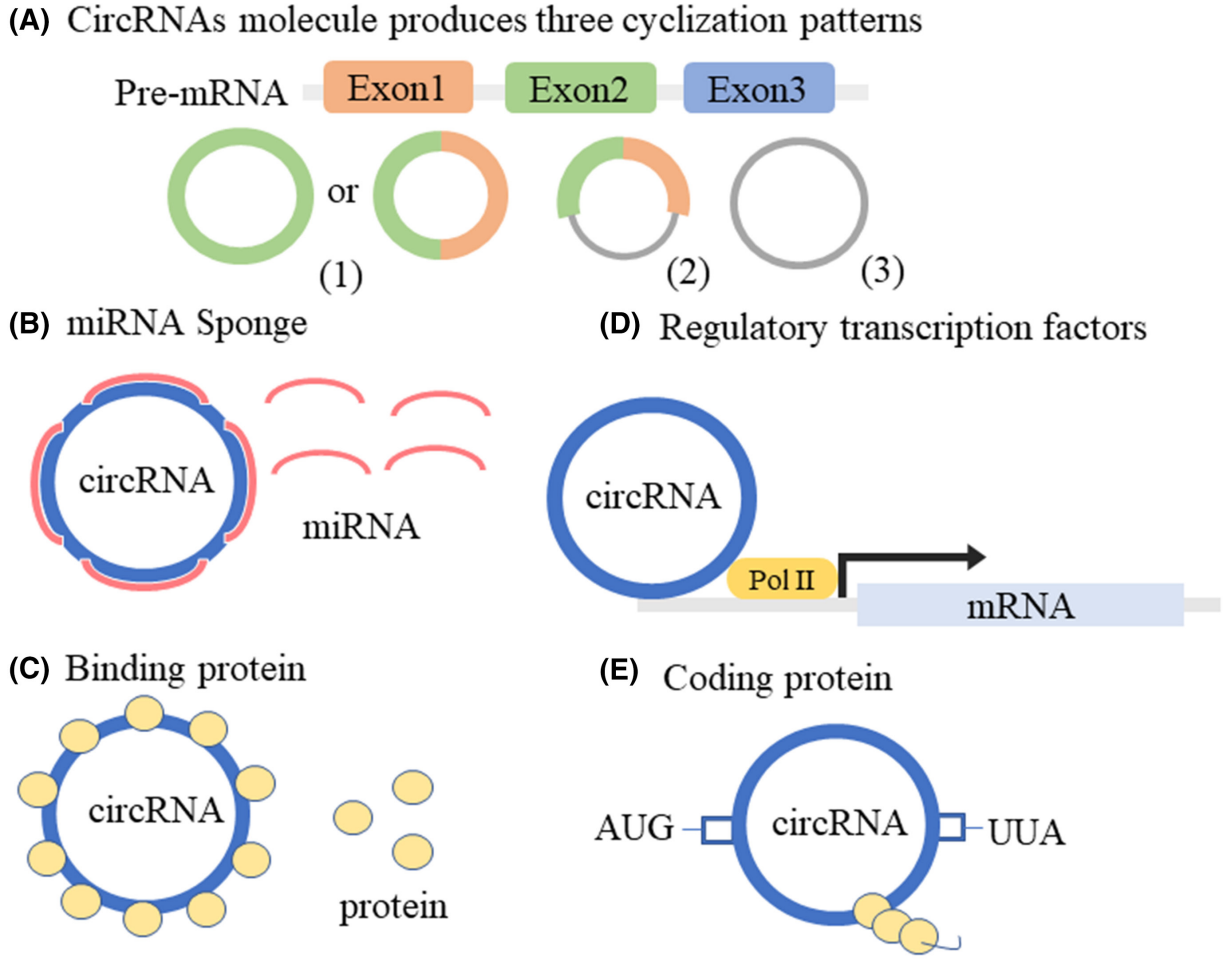
Molecular functions of circular RNAs (circRNAs). (A) CircRNA molecules produce three cyclization patterns: (1) exonic circRNAs, (2) exon‐intron circRNAs, and (3) intronic circRNAs; (B) CircRNAs act as miRNA sponges to regulate the activity of miRNA target genes; (C) CircRNAs bound to RNA binding proteins; (D) CircRNAs also directly participated in protein translation, affects protein‐coding function, and regulated transcription; (E) CircRNAs encoded peptides or proteins and affected their biological functions.

CircRNAs are a class of novel noncoding RNAs that are covalent closed‐loop structures without the 5′ caps or 3′ poly (A) tails that linear RNAs possess, forming a continuous ring structure through covalent bonds.^25^ CircRNA molecules have three cyclization patterns in different organisms: exon‐skipping or lariat‐driven circularization, direct back‐splicing or intron‐pairing‐driven circularization, and RNA‐binding protein‐driven circularization^26^, ^27^ (Figure 2A). CircRNAs were highly abundantly expressed in the brain^8^ and involved in the regulation of biological processes through various regulatory mechanisms. For example, circRNAs regulated the expression of target genes through microRNAs (miRNAs) (Figure 2B). Previous studies have found that *circRNA antisense to the cerebellar degeneration‐related protein 1 transcript* (*CDR1as*) had a powerful miRNA sponge effect and multiple miRNA‐binding sites, such as *miR‐7*, *miR‐135a*, and *miR‐876‐5p*.^28^, ^29^, ^30^ CircRNAs could also interact with specific RNA‐binding proteins, thereby affecting the expression of related proteins (Figure 2C). *CircRNA muscleblind* (*circMbl*) was generated by circularization of the second exon of the *Drosophila mbl* gene, which in turn regulated *circMbl* levels by binding to the Mbl produced by its native *mbl* gene.^31^ Another *circRNA glutamate ionotropic receptor AMPA type subunit 1* (*circGRIA1*) was abundantly expressed in the brain and could bind to glutamate receptor 1 to regulate synaptic plasticity and improve age‐related synaptic function.^32^ Furthermore, circRNAs play important roles in cell biology as transcriptional regulators (Figure 2D). For example, *circRNA human antigen R* (*circHuR*) interacted with CCHC‐type zinc finger nucleic acid binding (CNBP) to inhibit the binding of CNBP to the *HuR* promoter, thereby downregulating HuR expression levels and inhibiting the pathological process of gastric cancer.^33^ Finally, although circRNAs were noncoding proteins, some recently discovered circRNAs function as coding proteins (Figure 2E). *CircRNA zinc finger protein 609* (*circ‐ZNF609*), which encoded a protein, had an open reading frame, and the basic elements of start and stop codons were the same as those of linear transcripts.^34^ In summary, circRNAs mainly function by acting as miRNA sponges, regulating transcription, binding proteins, and translating polypeptides. CircRNAs, as a research hotspot in the field of ncRNAs, will reveal more important functions and mechanisms with the development of new technologies in the future.

### 3.2 Expression patterns of circRNAs in neurodegenerative diseases

CircRNAs are involved in the regulation of synaptic function, and their expression is age‐related and tissue‐specific. CircRNAs had a high expression level in the mammalian brain and accumulated in neuronal tissue with age,^8^, ^35^, ^36^ which might be due to their high stability.^37^ CircRNAs were highly conserved and specifically expressed in mouse, human, and *Drosophila* brain tissues.^8^, ^36^ Eighty percent of all circRNAs in mouse neurons with high expression levels were detected in the human brain.^8^ CircRNAs in the *Drosophila* brain were the most abundant among all tissues.^36^ The above results show that circRNAs have similar expression patterns in the brains of different model organisms, suggesting that circRNAs can stably exist in brain development and neuronal systems and can be used as biomarkers for brain pathologies.

Highly enriched circRNAs in the brain have extremely important roles in a variety of neurodegenerative diseases. Several dysregulated circRNAs have been identified in neurodegenerative diseases (Table 2). For example, 344 dysregulated circRNAs were detected in the brains of 6‐month‐old AD mice, of which 192 were upregulated and 152 were downregulated, and there were 244 dysregulated circRNAs in the brains of 9‐month‐old AD mice, of which 142 were upregulated and 102 were downregulated.^38^ CircRNAs are abundant and dynamically expressed, suggesting their role in neurodevelopment as well as in the pathogenesis and progression of neurological diseases.

**TABLE 2:** Overview of differentially expressed circRNAs in neurodegenerative diseases.

Diseases type	Sample	Number of circRNAs (up/down)	References
AD	Brains of 6‐month‐old AD mice Brains of 9‐month‐old AD mice	344 (192/152) 244 (142/102)	38
PD	Peripheral blood of PD patients	411 (129/282)	99
HD	HD mouse PC12 cell line model	23 (4/19)	13
ALS	Peripheral blood nuclear cells of ALS patients	521 (373/148)	96

Abbreviations: AD, Alzheimer's disease; ALS, amyotrophic lateral sclerosis; HD, Huntington's disease; PD, Parkinson's disease.

## 4 CIRCRNAS INVOLVED IN REGULATION OF NEURODEGENERATIVE DISEASES

CircRNAs could affect a variety of brain diseases, such as brain tumors, and acute and chronic neurodegenerative diseases, by affecting angiogenesis, neuronal plasticity, autophagy, apoptosis, and inflammation.^39^ At present, multiple circRNAs involved in neurological diseases by participating in important components of the synaptic system and presynaptic and postsynaptic neurons, thereby affecting the development and biogenesis of the nervous system, such as *circRNA regulating synaptic be exocytosis 2* (*circRims2*),^40^ *circRNA E74‐like ETS transcription factor 2* (*circElf2*),^41^ and *circRNA SHOC2 leucine‐rich repeat scaffold protein* (*circSHOC2*)^42^ (Table 3).

**TABLE 3:** Function of circRNAs in neurodegenerative diseases.

Diseases	CircRNA	Expression	miRNA	Targets	Samples	Function	Ref.
AD	*CDR1as /CiR‐7*	Down	*miR‐7*	UBE2A	AD patients	The expression of UBE2A was regulated by binding miR‐7 and amyloid peptide was eliminated	53
	*CircNF1‐419*	Up	—	Dynamin‐1 AP2B1	Male SAMP8 mice	Bound to dynamin‐1 and AP2B1 to eventually decrease levels of Tau and amyloid β‐protein	54
	*CircHDAC9*	Down	*miR‐138*	*ADAM10*	Male APP/PS1 transgenic mice	It promoted Aβ production, and induced synaptic and learning/memory deficits	56
	*CircHOMER1*	Down	*miR‐651*	*PSEN1/PSEN242*	AD female patients	It affected synaptic development	4, 57
	*CircCORO1C*	Up	*miR‐105*	*APP/SNCA*	AD patients	*CircCORO1C* reduced the accumulation of Aβ and SNCA in neurons	4
	*CircAβ‐a*	Up	—	—	HEK175 cells and human brain	It encoded Aβ175‐associated peptides (19.2 kDa)	62
	*CircCwc27*	Up	—	*Pur‐α*	AD patient brain and APP/PS1 transgenic mice	*CircCwc27* bound to *Pur‐α* inhibition to regulate APP proteins	59
PD	*CircSLC8A1*	Up	*miR‐128*	Ago2	Human brain tissues and Human neuroblastoma (SH‐SY5Y) cells	It regulated the oxidative activity of neurons and protects dopaminergic neurons from apoptosis	79
	*Circzip‐2*	Down	*miR‐60*	*zip‐2*	*C. elegans*.	It regulated the expression of the standard gene *zip‐2*, thereby affecting SNCA aggregation and reactive oxygen species content	6
	*CircSNCA*	Down	*miR‐7*	*SNCA*	*Strains of C. elegans*	*CircSNCA* increased *SNCA* expression by downregulating *miR‐7* in PD	68
	*CircSAMD4A*	Up	*miR‐29c‐3p*	MPTP MPP+	SH‐SY5Y cells	*CircSAMD4A* participated in the apoptosis and autophagy of dopaminergic neurons by modulating the AMPK/mTOR cascade via *miR‐29c‐3p* in PD	71
	*CircDLGAP4*	Down	*miR‐134‐5p*	*CREB* *Phospho‐CREB* *BCL‐2*	MPTP‐induced PD mouse model and MPP+‐induced PD cell models	*CircDLGAP4* exerted neuroprotective effects via modulating *miR‐134‐5p*/*CREB* pathway	72
	*CircTLK1*	Up	*miR‐26a‐5p*	*DAPK1*	MPTP‐induced mouse model and rotenone‐ and MPP + −induced cell model	Depletion of *circTLK1* mitigated dopaminergic neuron injury in vitro and in vivo, via releasing *miR‐26a‐5*p to target *DAPK1* expression	76
	*Circ_0070441*	Up	*miR‐626*	*IRS2*	MPP‐treated SH‐SY5Y cells	*Circ_0070441* aggravated MPP+ triggered neurotoxic effect in SH‐SY5Y cells by regulating *miR‐134‐5p q*/*IRS2* axis	78
HD	*CircHTT*	Up	—	—	HEK293 and SH‐SY5Y cells	It decreased cell proliferation, nuclear area, and altered subcellular localization of the HTT protein	92
ALS	*Circ‐Hdgfrp3*	—	—	—	Murine HBG3 ES cells Generation of embryoid bodies Murine Neuro‐2a cells Human SK‐N‐BE cells	Mutant FUS protein (mtFUS) affected the localization of circ‐Hdgfrp3 under oxidative stress conditions	96

Abbreviations: AD, Alzheimer's disease; ALS, amyotrophic lateral sclerosis; HD, Huntington's disease; PD, Parkinson's disease.

### 4.1 CircRNAs in AD

**FIGURE 3:**
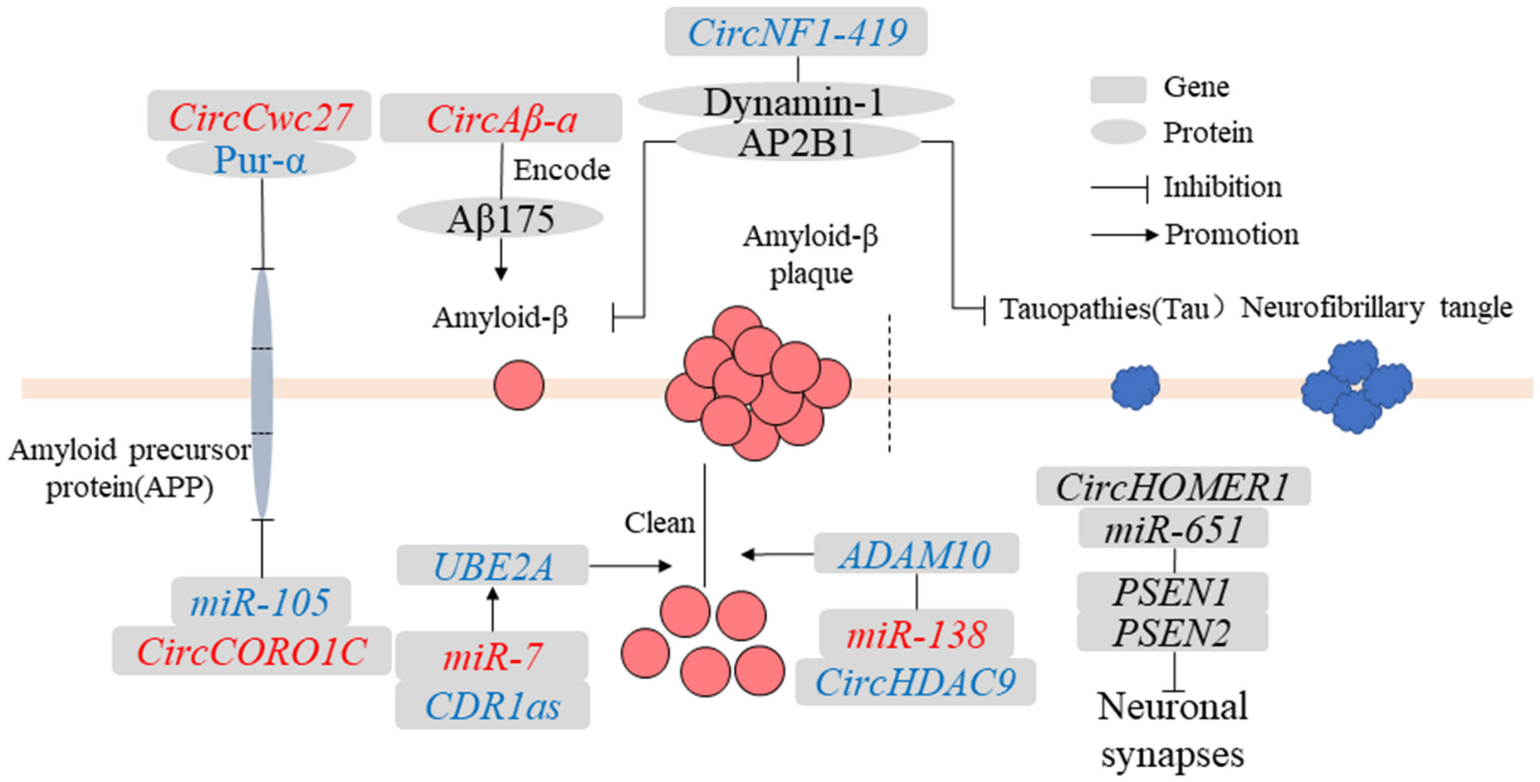
Regulatory mechanisms of circRNAs in AD. Red words indicate the upregulated noncoding RNAs and blue words indicate the downregulated noncoding RNAs. CircRNA, circular RNA; miR, microRNA.

AD is a neurodegenerative disease typically of insidious onset and characterized by the presence of neurotic plaques and neurofibrillary tangles.^43^ AD had two basic pathological features: (1) interneuronal neurofibrillary tangles composed of abnormally modified tauopathies and (2) accumulation of Aβ and formation of senile plaque deposits.^44^ In addition, inclusion bodies of TDP‐43 protein had also been found in AD patients.^45^, ^46^ To date, several circRNAs have been found to play a role in the regulation of AD pathological processes (Figure 3).

#### 4.1.1 *CDR1as*

*CDR1as* was currently the most studied circRNA and was involved in various cancer pathologies and neuronal phylogeny.^47^, ^48^, ^49^ *CDR1as* had a powerful miRNA sponge function and regulated the levels of target genes by adsorbing miRNAs.^50^ Multiple *miR‐7* binding sites were found on *CDR1as*, which could increase the expression of the downstream target gene *ubiquitin‐conjugating enzyme E2A* (*UBE2A*).^50^ UBE2A typically coordinated the clearance and degradation of amyloid and damaged proteins by the proteolysis of 26S proteasomes during the ubiquitination cycle.^51^, ^52^ In sporadic AD brains, the regulation of the ubiquitination cycle involves a genetic defect that may lead to the inability to clear Aβ peptides from the cytoplasm.^53^ Therefore, the study of the miRNA sponge function of circRNAs is a key approach to the important epigenetic regulatory mechanisms of circRNAs in the central nervous system pathogenic gene expression program.

#### 4.1.2 CircNF1‐419

*CircRNA neurofibromin 1‐419* (*circNF1‐419*) derived from the *neurofibromin 1* (*NF1*) gene, bound to dynamin‐1 and adaptor protein 2 B1(AP2B1) proteins in the neonatal rat cerebral cortex.^54^ Dynamin‐1 and AP2B1 proteins regulated autophagy activity through PI3K‐I/Akt‐AMPK‐mTOR and PI3K‐I/Akt–mTOR signaling pathways to affect the levels of aging markers (p21, p35/25, and p16) and inflammatory factors (tumor necrosis factor‐α and NF‐κB), which reduced the expression of AD marker proteins tau and Aβ, thereby delaying the pathological process of AD.^54^ The process of aging was often accompanied by the pathological processes of neurodegenerative diseases and many related features of aging that have been found in neurodegenerative diseases.^55^ Thus, *circNF1‐419* affects the AD marker protein levels through age‐related signaling pathways.

#### 4.1.3 *CircHDAC9*

*CircRNA histone deacetylase* (*circHDAC9*) in the mouse hippocampus had multiple binding sites for *miR‐138* to promote the expression of the target gene *sirtuin 1* (*Sirt1*), which increased the content of Aβ and further led to synaptic and learning/memory deficit damage in neuronal function.^56^ The expression level of *circHDAC9* was decreased in the serum of patients with AD and those with mild cognitive impairment.^56^ These results suggest that *circHDAC9*/*miR‐138*/*Sirt1* plays an important regulatory role in synaptic dysfunction and abnormal Aβ splicing, providing a new therapeutic target for AD patients.

#### 4.1.4 *CircHOMER1*

*CircRNA homer protein homolog 1* (*circ HOMER1*) was produced by alternative splicing of the *HOMER1* gene. The linear and circular expression levels of *HOMER1* were significantly downregulated in the development of prefrontal cortex and pluripotent stem cell‐derived neurons in patients with schizophrenia and bipolar disorder.^57^ The *HOMER1* was involved in synaptic plasticity, learning, and memory, and affected Aβ deposition in the cerebral cortex.^57^ Furthermore, *circHOMER1* had multiple binding sites for *miR‐651*, targeting *presenilin‐1* (*PSEN1*) and *presenilin‐2* (*PSEN2*) genes to affect the development of neuronal synapses.^4^ Further research needs to determine how *circHOMER1* affects the expression of its native gene, *HOMER1*.

#### 4.1.5 *CircCORO1C*

C*ircRNA actin‐binding protein 1C* (*circCORO1C*), derived from *actin‐binding protein 1C* (*CORO1C*), had multiple *miR‐105*‐binding sites.^58^ *CircCORO1C* targeted *AD‐related genes β‐amyloid precursor protein* (APP) and *SNCA* by acting as a sponge for *miR‐105*.^4^ *APP* and *SNCA* were important risk factors for the course of AD.^58^ *CircCORO1C* reduced the accumulation of Aβ and SNCA in neurons and alleviated the pathological process of AD.^4^

#### 4.1.6 *CircCwc27*

*CircRNA spliceosome‐associated cyclophilin* (*circCwc27*), derived from the *CWC27* gene, was highly expressed in neurons, upregulated in the temporal cortex and plasma of APP/PS1 mice and AD patients and has an impact on cognition, neuropathology, and transcriptome in APP/PS1 mice.^59^ *CircCwc27* bound to Pur‐α in the cytoplasm.^60^ The interaction between Pur‐α and *circCwc27* was significantly enhanced in APP/PS1 mice, altering the transcription of AD‐associated genes and APP proteins.^60^ Pur‐α was involved in brain development, synaptic plasticity, and memory retention and played a key role in gene transcription regulation.^61^ Knockdown of *circCwc27* reduced the expression level of APP and the production of Aβ.^59^ Thus, circRNAs could directly affect the regulation of AD‐related pathological proteins and become promising AD therapeutic targets with clinical transformation potential.

#### 4.1.7 CircAβ‐a

*CircAβ‐a*, derived from the Aβ coding region of the *APP* gene, was detected in the brains of AD patients and nondementia control groups, and encoded a polypeptide (19.2 kDa) associated with Aβ175.^62^ This was a new method to synthesize Aβ proteins from circRNAs. Biogenesis of *circAβ‐a* did not require this specific mutation, unlike a specific mutation in the *APP* gene during AD pathology.^63^ It was likely that all human individuals produced *circAβ‐a*, suggesting that it may play a role in the pathogenesis of sporadic AD and that the translation of circRNAs could be activated and controlled under certain conditions.^34^, ^63^, ^64^ The Aβ ratio of *circAβ‐a* translation Aβ175 processing to APP full‐length protein‐derived peptides might be an important indicator for diagnosing AD pathology.

#### 4.1.8 The other circRNAs‐competitive endogenous RNA in AD

The multiple differentially expressed circRNAs had been identified in the hippocampus of AD model APP/PS1 mice, which were involved in the Hippo, cGMP‐PKG, and cAMP signaling pathway, affecting functions such as axon guidance and platelet activation in neuronal synaptic systems.^5^, ^38^ A variety of differentially expressed circRNAs act on sponges of miRNAs to regulate target genes. For example, both *mmu_circ_0001125* and *mmu_circ_0000672* regulated the expression of *cystatin F* (*Cst7*) by binding *mmu‐miR‐351‐5p*.^5^ As an endosomal/lysosomal cathepsin inhibitor, *Cst7* is involved in cathepsin activity in the lysosomal pathway, and reduced the phagocytic ability of activated microglia to promote clearance of Aβ.^65^ Therefore, the ceRNA regulatory network is an important epigenetic data of AD pathology, suggesting that various dysregulated circRNAs in AD pathology can serve as important biomarkers and therapeutic targets for neurodevelopment.

### 4.2 CircRNAs in Parkinson's disease

**FIGURE 4:**
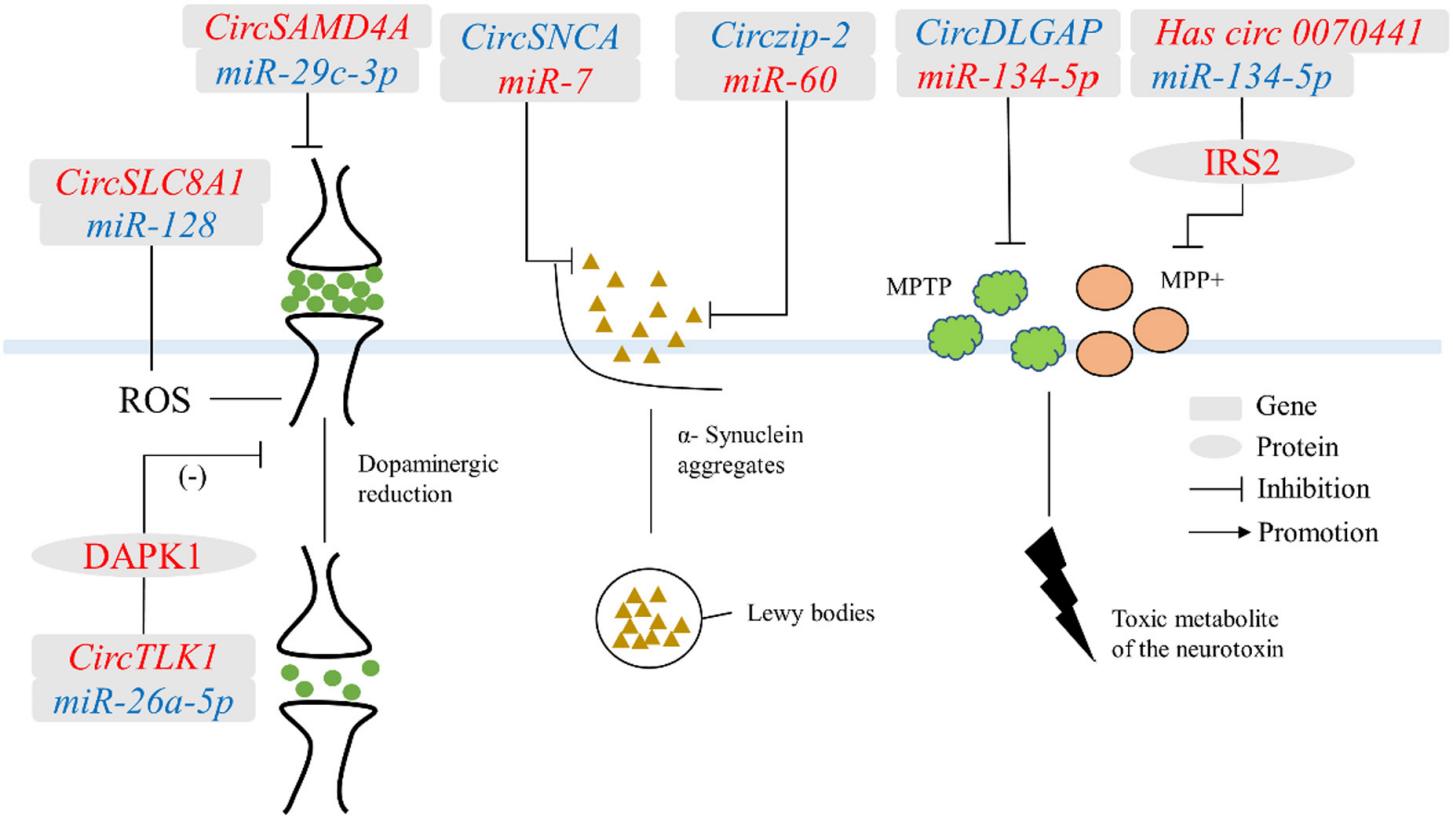
Regulatory mechanisms of circRNAs in Parkinson's disease. Red words indicate the upregulated noncoding RNAs and blue words indicate the downregulated noncoding RNAs. DAPK1, death‐associated protein kinase 1; circRNA, circular RNA; miR, microRNA; MPTP, 1‐methyl‐4‐phenyl‐1,2,3,6‐tetrahydropyridine; MPP+, 1‐methyl‐4‐phenylpyridinium; ROS, reactive oxygen species.

PD was a common progressive neurodegenerative disorder that was characterized by tremors and bradykinesia.^66^ The main pathological feature of PD was the degeneration of the Lewy body or the Lewy nerve synapses due to SNCA aggregation, resulting in the formation of filamentous cytoplasmic inclusions, accompanied by lesions in the substantia nigra and dysregulation of dopamine homeostasis.^67^ Most circRNAs regulate PD through a miRNA mechanism (Figure 4).

#### 4.2.1 Circzip‐2

*CircRNA zinc‐regulated transporters and iron‐regulated transporter‐like protein‐2* (*Circzip‐2*), derived from *zip‐2* genes, was significantly downregulated in the PD model of *C. elegans,* and formed a competitive inhibitory cleavage with its parental gene *zip‐2*.^6^ The zip‐2 protein directly affected the expression level of SNCA and the content of reactive oxygen species.^6^ In addition, *circzip‐2* mitigated the pathological process of PD by binding to *miR‐60*. The protective mechanism of *circzip‐2* against PD could be applied to the design of specific therapeutic molecules and the development of effective diagnostic tools for neurodegenerative diseases.

#### 4.2.2 *CircSNCA*

*CircRNA alpha‐synuclein* (*circSNCA*), sourced from the *SNCA* gene was downregulated in the 1‐methyl‐4‐phenylpyridinium (MPP+)‐treated SH‐SY5YPD cell model. *CircSNCA* regulated the expression of *SNCA* mRNA by binding to *miR‐7*.^68^ SNCA protein was related to neurotoxicity and anti‐apoptotic pathways and was the most important protein in PD pathological abnormal protein deposition. Therefore, downregulation of *circSNCA* could affect the expression of *SNCA* mRNA in the native gene, thereby reducing neuronal apoptosis and inducing autophagy in PD patients.^68^

#### 4.2.3 *CircSAMD4A*

*CircRNA sterile alpha motif domain containing 4A* (*circSAMD4A*) originated from *SAMD4A* and promoted apoptosis and autophagy.^69^ 1‐Methyl‐4‐phenyl‐1,2,3,6‐tetrahydropyridine (MPTP) and MPP+ were neurotoxins that affected the pathological process of PD.^70^ Human neuroblastoma cells (SH‐SY5Y) were treated with MPTP and MPP+ to establish a PD cell model.^70^ In this model, *circSAMD4A* regulated the levels of MPTP and MPP+ and participated in the apoptosis and autophagy of dopaminergic neurons through the AMPK/mTOR pathway^71^. Furthermore, the binding of *miR‐29c‐3p* to *circSAMD4A* attenuated the cytotoxicity of MPTP or MPP+ in dopaminergic neuronal cells^71^. Therefore, *circSAMD4A* might be as a promising diagnostic biomarker and therapeutic target for PD.

#### 4.2.4 *CircDLGAP*

*CircRNA DLG‐associated protein 4* (*circDLGAP4*) expression was decreased in MPTP‐induced PD mouse model and MPP^+^‐intoxicated PD cell models. In vitro, *circDLGAP4* might be promoted in the development of PD by affecting SH‐SY5Y and MN9D cell viability, apoptosis, mitochondrial damage, and autophagy.^72^ In addition, *circDLGAP4* exerted its function by regulating *miR‐134‐5p*.^73^ *CircDLGAP4*/miR‐134‐5p also regulated the activation of *CREB* signaling and the expression of *CREB* target genes.^73^ *CircDLGAP4*/*miR‐134‐5p*/*CREB* axis could explain the pathogenesis of PD in human and mouse models.

#### 4.2.5 *CircTLK1*

*CircRNA tousled‐like kinase 1* (*CircTLK1*), derived from the *TLK1* gene, regulated tumor cell proliferation, metastasis, and myocardial ischemia/reperfusion injury.^74^, ^75^ Then, *circTLK1* was significantly increased in MPTP‐induced PD mouse models.^76^ Knockdown of *circTLK1* inhibited apoptosis and toxicity in PD pathological mouse model cells and improved cell viability.^76^ In addition, *circTLK1* acted as a *miR‐26a‐5p* sponge to regulate its target gene [*death‐associated protein kinase 1* (*DAPK1*)] and improved neurological dysfunction caused by middle cerebral artery occlusion and reperfusion in vivo.^76^ The *circTLK1/miR‐26a‐5p/DAPK1* regulatory axis highlights the role of *circTLK1* in the pathogenesis of PD and provides a new theoretical basis for the development of effective treatments for PD.

#### 4.2.6 Circ_0070441

*Circ_0070441* originated from the *SNCA* gene, and its expression was upregulated in the PD cell model.^68^ In a PD model constructed with MPP + ‐treated SH‐SY5Y cells, *circ_0070441* upregulated the expression level of the target gene *insulin receptor substrate 2* (*IRS2*) by adsorbing *miR‐626*. ^77^The IRS2 protein is involved in the pathological process of PD through affecting the Aβ level and reducing the cellular neurotoxins. ^78^In the regulatory mechanism of PD, the ceRNA network involved in c*irc_0070441* may provide a new target for PD treatment.

#### 4.2.7 *CircSLC8A1*

*CircSLC8A1* (*solute carrier family 8 member A1*) was increased in cultured cells, and exposed to the oxidative stress‐inducing agent paraquat.^79^ *CircSLC8A1* had seven binding sites for *miR‐128* and is strongly bound to the microRNA effector protein Argonaute 2(Ago2).^79^ Three target genes (*silent information regulator transcript1* [*SIRT1*], *B cell‐specific Moloney murine leukemia virus integration site 1* [*BMI1*], *Axis inhibition proteins 1* [*Axin1*]) of *miR‐128* played an important regulatory role in neuronal denaturation, neuronal oxidative activity, and protection of dopaminergic neurons from apoptosis.^80^, ^81^, ^82^ Oxidative stress is considered an important cause of many neurodegenerative disorders. Thus, *circSLC8A1* can regulate oxidative stress activation, which has great significance in the pathology of PD.

### 4.3 CircRNAs in HD

HD is a dominantly inherited neurodegenerative disease caused by repeated expansion of the CAG nucleotide sequence in the *Huntington* gene on chromosome 4, resulting in abnormal gene expression. Mutant huntingtin (mHTT) produced abnormally long polyglutamine repeats.^83^ Twenty‐six or fewer repeat amplifications of CAG were considered normal, whereas 27–35 replicates posed a risk of spreading diseases secondary to dilation through parental meiosis. Furthermore, 36–39 replicated CAG represented incomplete penetrance of HD pathology peaks, whereas CAG‐expanded alleles beyond 40 repeats were considered fully penetrant genes and caused HD.^84^ More CAG repeat expansions were generally associated with an earlier age of onset, and male sperm had a greater potential for repeat variation. Therefore, HD was often associated with genetic predisposition in men.^85^, ^86^ In addition, mHTT proteins lead to neuronal dysfunction and death through various mechanisms, such as the regulation of cellular protein stabilization, axonal transport, transcription, translation, and mitochondrial and synaptic functions.^87^, ^88^, ^89^

One study found 19 downregulated and 4 upregulated circRNAs between mouse PC12 cell lines expressing wild‐type huntingtin protein as a control and mHTT protein.^90^ In this study, 16 downregulated circRNAs came from the same chromosomal region of the *Rere* gene, and the remaining three downregulated circRNAs came from other chromosomal regions.^90^ From the analysis of the mechanism, 15 of the 23 differentially expressed circRNAs were related to the MAPK pathway, and 16 were involved in the dopaminergic synaptic pathway. The pathological mechanism of dopamine in HD has been widely demonstrated.^90^ The MAPK pathway had important effects on cell proliferation and division, the stress response, differentiation, and apoptosis; c‐Jun N‐terminal kinase (JNK) and p38 in the MAPK pathway were the main signaling factors involved in the pathogenesis of HD.^91^ In summary, circRNAs might regulate the pathological process of HD through dopaminergic synapses and MAPK pathways, and their specific biological functions require further exploration.

Another study found that *circHTT 2–6* derived from the exons 2, 3, 4, 5, and 6 of the *HTT* gene was enriched in in the frontal cortex of HD patients.^92^ When *circHTT* (2–6) was overexpressed in HEK293 and SH‐SY5Y cells, no change in the CAG repeat region of HD was detected, which decreased the cell proliferation, nuclear area, and altered subcellular localization of the HTT protein.^92^ These results demonstrate the overexpression of *circHTT* undergoes HD‐related pathological changes, but its specific functional mechanism needs further study.

### 4.4 CircRNAs in ALS

ALS is a heterogeneous neurodegenerative disease whose main pathological features are the degeneration of upper motor neurons that project to neurons in the brainstem and spinal cord and lower motor neurons that the brainstem or spinal cord projects onto muscles. ALS patients had features of TDP‐43 proteinopathy, such as loss of TDP‐43 in the nuclei of neuronal cells and cytoplasmic aggregated with skeletal‐like or dense morphology in residual motor neurons.^93^

In addition, circRNAs have been involved in the regulation of fused in sarcoma (FUS) proteins during ALS.^94^ For example, *circ‐Hdgfrp3* took part in protecting neuronal function and integrity in neuronal cells, whereas mutant FUS protein (mtFUS) affected the localization of *circ‐Hdgfrp3* under oxidative stress conditions.^95^ The mtFUS could lead to abnormal accumulation of cytoplasmic FUS protein and increased mitochondrial translocation, resulting in excessive mitochondrial fission and damage, eventually leading to neuronal death, which was a major pathological feature in some ALS patients.^12^ In summary, circRNAs are likely to be involved in the pathological regulation of ALS.

Many dysregulated circRNAs were also found in ALS patients, including 151 downregulated circRNAs, most of which were involved in the pathological process of ALS.^96^ *Hsa_circ_0000567* derived from the *SETD3* gene, and SETD3 was the histone methyltransferase that regulated muscle differentiation in mouse.^97^ *Hsa_circ_0023919* originated from the *PICALM* gene, which was involved in clathrin‐mediated endocytosis at neuromuscular junctions. Single‐nucleotide polymorphisms upstream of the gene were associated with AD.^98^ Simultaneously, the authors evaluated the correlation between the expression levels of circRNAs and the potential association with clinical data.^96^ Three circRNAs (*hsa_circ_0000567*, *hsa_circ_0023919*, and *hsa_circ_0088036*) were negatively correlated with patient age at the time of blood collection.^96^ The sensitivity and specificity of these three circRNAs were as high as 90% in patients with ALS, significantly higher than the most representative biomarkers in ALS, phosphorylated neurofilament heavy chain and neurofilament light chain.^96^ Therefore, circRNAs have great potential as biomarkers for ALS diagnosis and treatment.

## 5 CONCLUSION

The brain is the most plastic organ, and its circuits are tightly regulated and modified throughout an organism's lifespan. The high abundance of circRNAs in the brain indicates that they are involved in the regulation of the nervous system. The diversity of causative factors in neurodegenerative diseases makes blocking one or both pathways incapable of significantly reducing overall neuronal dysfunction and loss. With the continuous deepening of research on neurodegenerative diseases, multi‐channel and multi‐targeted treatments can improve the symptoms of patients, regulate brain function, and play a therapeutic role. However, the course of neurodegenerative diseases often involves in cognitive impairment in the middle and late stages when treatment can only slow down the development of the disease and cannot fundamentally reverse the damage to neural networks. Therefore, the high stability and tissue specificity of circRNAs make them an important pathological detection marker, which has important guiding significance for the early treatment and diagnosis of neurodegenerative diseases as a key biomarker.

Current research mainly focuses on circRNAs in the neurodegenerative diseases AD and PD, and research on circRNAs in other neurodegenerative diseases needs to be supplemented. However, the powerful role of circRNAs in AD and PD is likely to be important in other pathologies. Further studies on circRNA structure and function will improve our understanding of the pathogenesis of neurological diseases and lead to the development of new diagnostic and treatment methods.

## AUTHOR CONTRIBUTIONS

Contributions: Feng Xiao and Deying Yang concept and design, literature search, manuscript preparation, manuscript editing; Jiamei Li, Siqi Wang: literature search, manuscript editing and review; Deying Yang, Zhi He: conceptual design, writing guidance, manuscript review; Mingyao Yang, Xiaolan Fan: conceptual design, directed review; Taiming Yan: manuscript review; Feng Xiao, Deying Yang edited the manuscript, and all authors approved the final version of the review.

## FUNDING INFORMATION

The study was supported by the National Natural Science Foundation of China (grant numbers 31972777, 2019; 31402286, 2015) and the China Scholarship Council (202106915017).

## CONFLICT OF INTEREST STATEMENT

The authors declare no conflicts of interest.

## Data Availability

Data sharing not applicable to this article as no datasets were generated or analysed during the current study.
